# Characteristics of picky eater children in Turkey: a cross-sectional study

**DOI:** 10.1186/s12887-022-03458-0

**Published:** 2022-07-20

**Authors:** Suzan Yalcin, Ayse Oflu, Mustafa Akturfan, Siddika Songul Yalcin

**Affiliations:** 1 Department of Food Hygiene and Technology, Faculty of Veterinary Medicine, Selcuk University, Konya, Turkey; 2 Department of Pediatrics, Afyonkarahisar Health Sciences University Faculty of Medicine, Afyonkarahisar, Turkey; 3 Department of Hotel, Restaurant and Catering, Karamanoğlu Mehmetbey University Vocational School of Social Sciences, Karaman, Turkey; 4 Department of Pediatrics, Faculty of Medicine, Hacettepe University, Ankara, Turkey

**Keywords:** Picky eating, Parent, Sleep, Screen, Physical activity

## Abstract

**Objective:**

The aim of this study is to investigate the relations of picky eating habit of children with their nutrition, physical activity, screen time and sleep habits in the context of parental picky eating habit.

**Methods:**

In a cross-sectional study a questionnare was applied to the parents of children aged 6–13 years in two provincies. The cases were analyzed as the overall group and the two subgroups in which both parents are not picky eater, and in which at least one parent was picky eater. Child's risk of being picky eater was analyzed by logistic regression.

**Results:**

A total of 913 children and parent pairs were included in the study. The risk of picky eating of the child increases 2.85 (AOR: 1.67–4.88) times when only the mother was picky eater, 5.99 (AOR: 3.32–7.52) times when only the father was picky eater, and 22.79 (AOR: 6.95–74.71) times when both of the parents were picky eaters. In the subgroup in which at least one parent was picky eater, it was determined that children with physical activity duration of ≥ 1 h/day and sleep time of ≥ 9 h /day were less likely to be picky eater and the children with screen time of ≥ 2 h/day were more likely to be picky eaters.

**Conclusion:**

Picky eating habit in childhood is related to the picky eating habit of the parents. In the context of this relationship, the picky eating habit of children is related to sleep, physical activity, screen time and other eating habits.

## Introduction

Picky eating (PE) behavior is particularly common in early childhood and challenging for parents [1]. Although there is no formal definition, the most widely accepted definition of PE was proposed by Dovey et al. as children who “consume an insufficient variety of food while rejecting significant amounts of food they are familiar and also unfamiliar” [2]. In addition to this definition, it includes problematic issues such as limited vegetable consumption, excessive food preference, choice of meals different from caregivers’ and special food preparation methods [3]. The prevalence of this problem, which has led to such negative consequences, is currently confused due to the lack of a complete definition and methodological differences in researches [4]. Today, a prevalance varying 13% to 47% in school-age children has been reported [1, 4].

Although there are many studies on picky eater children, the number of studies investigating the parental characteristics of these children is limited. A recent study showed that caregivers of children who are picky eaters were more likely to perceive their children as underweight than caregivers of non-picky eater children [5]. Another study showed that parents with low fruit and vegetable consumption were more likely to use pressure on their children to increase their food intake, whereas children who were forced to “eat their vegetables” consumed less fruit and vegetables [6].

Generally, PE can lead to lower intake of vitamins, minerals, whole grain products and dietary fiber, while increasing the risk of being underweight/thinness or overweight [7]. Studies showed that PE is also associated with behavioral problems and symptoms of anxiety and depression [8, 9] and it may turn into an eating disorder in adolescence and adulthood [4]. PE was also found to be related with overweight and obesity in children [10, 11]. Decrease in food fussiness during a family-based obesity intervention has been associated with greater weight reduction with preschool children [12]. This suggests that PE can lead to overweight and obesity. While the previous literature did not show a relationship between PE behavior and obesity-related eating behaviors in children, it has found that sedentary lifestyle behaviors are more common in children with PE. Parents of picky eaters also reported that their children had too much screen time but complained about physical activity [13]. In addition, although the relationship between PE and sleep habits was not investigated in previous studies, it has been reported that healthy sleep behaviors and healthy eating habits are associated [14].

The aim of this study is to determine the prevalance of PE among Turkish school aged children and the assosications of PE habit of children with the independent variables as socio-demographic characteristics and PE status of the parents and with the dependent variables as antropometric measures, physical activity, screen time, sleep time, dietary habits of children. Knowing the individual and parental characteristics of children who are picky eaters can help doctors guide the parents in raising healthy children.

## Method

### Study design

This study was designed as a cross-sectional survey and carried out in two provinces (Afyonkarahisar and Konya) between December 1, 2018 and January 15, 2019 in Turkey. Afyonkarahisar is a small-scale province located in the western region of Turkey, while Konya is a large-scale province located in the inner region. The ratio of the child population to the total population is 26% in Afyonkarahisar and 28% in Konya, which is close to the Turkish average of 27.2% [15]. The study was conducted on children aged 6–13 years attending a total of four schools; one primary and one secondary school from each city. These schools were randomly selected from schools that could represent students of different sociodemographic characteristics, with the recommendation of the Research and Development Unit of the Directorate of National Education. Permission to carry out the study was approved by Hacettepe University’s Clinical Research Ethics Committee. All study procedures were performed in accordance with the Declaration of Helsinki.

### Study population and sample size

Sample size was calculated as 384 when the parameters were sustained as 1,000,000 for population size, 50% [16] for the frequency of picky eating among school aged children in the population, 5% for confidence limits and 1 for design effect [17]. Considering gender (male and female) differences, it was taken as 768. For the possibility of 25% disagreement, 960 child-parent couples were invited to participate in the study. Children with chronic diseases affecting their eating habits and growth status were excluded from the study.

### Study protocol and study file

After the children were informed about the purpose and procedures of study, the study file and consent forms were distributed to children. Written informed consent was obtained from all the participating parents and verbal consent was obtained from all the children. Then the study files were filled out by the parents and the forms were collected the next day. If more than one child in the family was eligible for the study, only the oldest sibling from each family were included in the study. The study file was created by the authors and the adequacy of the questions was checked with a preliminary study, then study file was finalized. It consisted of three categories and a total of 30 questions. In the first section, sociodemographic and anthropometric characteristics, in the second section lifestyle habits and in the third section dieatry habits were taken [16, 18]. The variables of the study are as follows: PE status of children as dependent variable and the socio-demographic characteristics of family, PE status of the parents, children’s height and weight, physical activity time, screen time, sleep time, bedtime and dietary habits (having breakfast, skipping meals, mealing at table, mealing in front of screen, snacking at night, having sugary snacks, having salty snacks, drinking sugary-carbonated beverage, drinking ayran) as independent variables.

We defined the picky eater as ‘‘someone who refuses familiar (and unfamiliar) foods and/or consumes insufficient variety and quantity of food consumed in the family's home environment’’ [2] in the study file and the parent’s perceptions were used to decide whether their child was a “picky eater”. Then, the participating parents (usually mother) were asked a single question, ‘‘Are you/your spouse/child picky eaters?’’ Parent marked one of the yes or no options for themself, their spouse and children. If they chose “Yes” for one, parent or child was considered as a picky eater.

Body mass index (BMI) was calculated as [weight in kilograms divided by the square of the height in meters (kg/m^2^)]. BMI for age z-scores (BAZ) was determined using the age and gender specific Growth Standards using World Health Organization (WHO) antro-plus software [19]. Children with BAZ < -2 were considered as underweight (thinness), (-2)-(+ 1) as normal weight, (> + 1)-(+ 2) as overweight and >  + 2 as obese based on the guidelines of the WHO. Physical activity habit of children was classified; < 60 min/day as under than recommended, ≥ 60 min/day as recommended, based on daily activity times [20]. Screen time of children was classified; < 2 h/day as recommended, ≥ 2 h/day as higher than recommended [21]. Sleep habits of children were evaluated as daily sleep time and bedtime. Daily sleep time was assessed; < 9 h/day as insufficient; 9–12 h/day as adequate; > 12 h/day as excessive [22]. Bedtime was classified as 10:00 pm and before as age appropriate, after 10:00 pm as late [23]. In order to examine dietary habits of children; mealtime of children, habits of having breakfast, skipping meals, mealing at table, mealing in front of screen, snacking at night, having sugary snacks, having salty snacks, drinking sugary-carbonated beverage, drinking ayran were questioned. Mealtime of children was classified; < 20 min as quick; 20–30 min as normal, > 30 min as slow [24].

### Statistical analysis

The data were analyzed using the SPSS package program 22.0 and evaluated using descriptive statistics; arithmetic mean, standard deviation, and percentage distributions. Chi-square test was used when comparing percentage distributions of categorical data between the groups. Child’s risk of being picky eater according to the parents’ status of being picky eater was calculated by logistic regression. The cases were analyzed as (a) the overall group and the two subgroups (b) in which both parents are not picky eater, and (c) in which at least one parent was picky eater. The picky eater status of the children according to the cases’ characteristics was analyzed by chi-square test in all three groups. Adjusted residuals were used to determine the subgroup differences in 3X2 groups. Multivariable logistic regresson analysis was used to evaluate the association between child’s PE status and selected characteristics of mother–child pairs such as the gender and age of the children, the number of children, mother’s age, mother’s education, mother’s occupation, mother’s perception of the child’s weight in overall group. Adjusted Odds Ratio (AOR) and 95% confidence intervals (CI) were calculated. The same analysis was also repeated in the subgroups, in which both parents are not picky eater, and in which at least one parent was picky eater. By adjusting the general characteristics of the child-mother couple, the children’s risk of being picky eater according to each of the children’s lifestyle habits was tested with multivariable logistic regresson analysis in overall group and subgroups. *P* values < 0.05 were considered as statistically significant.

## Results

### General characteristics

**Fig. 1:**
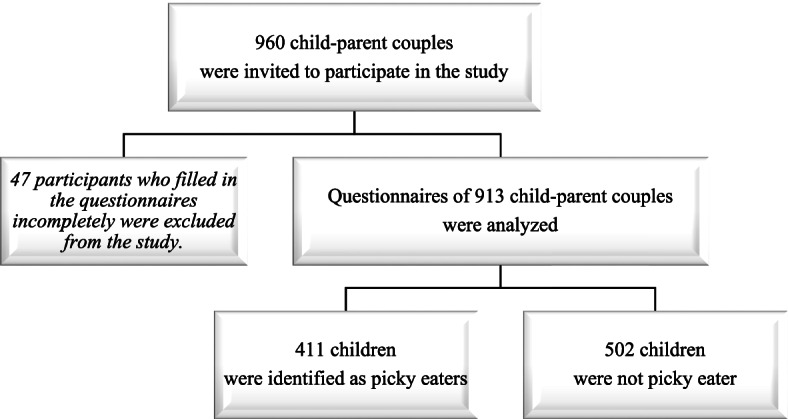
Flowchart of the study

A total of 913 child-parent pairs were included in the study. Figure 1 shows the flowchart of the study. The mean age of the children was 9.8 ± 2.0 years and (range: 6–13 years). According to the age range of children going to primary and secondary school, children were categorized as 6–9 years old and 10–13 years old in terms of age. The mean age of the mothers was 37.2 ± 5.2 years (range: 24–62) and the mean age of the fathers was 39.1 ± 5.5 (range: 25–67) years. The frequency of PE in children was determined as 45.0% and it was found to be 11.1% in mothers and 19.4% in fathers. Table 1 shows the comparison of general characteristics of all participant children with parental picky eating status. When at least one picky eater parent were present in family, mothers most likely perceived her child as overweight (*p* < 0.001).

**Table 1:** Baseline characteristics of enrolled child and parents and comparisons for characteristics according to parental picky eating status

		**Parental picky eating status**		
	**Overall (*n* = 913)**	**None (*n* = 675)**	**At least one (*n* = 238)**	
	n(%)^c^	n(%)^c^	n(%)^c^	p
**Gender**				
Female	492 (53.9)	359 (53.2)	133 (55.9)	0.473
Male	421 (46.1)	316 (46.8)	105 (44.1)	
**Age group**				0.297
6–9 years	476 (52.1)	345 (51.1)	131 (55.0)	
10–13 years	437 (47.9)	330 (48.9)	107 (45.0)	
**Number of children in the family**				0.122
1	90 (9.9)	60 (8.9)	30 (12.6)	
2–3	707 (77.4)	523 (77.5)	184 (77.3)	
≥ 4	116 (12.7)	92 (13.6)	24 (10.1)	
**Age of mother at birth of enrolled child**				0.114
< 25 years	299 (32.7)	214 (31.7)	85 (35.7)	
25- < 30 years	372 (40.7)	270 (40.0)	102 (42.9)	
≥ 30 years	242 (26.5)	191 (28.3)	51 (21.4)	
**Age of father at birth of enrolled child**				0.050
< 25 years	173 (18.9)	127 (18.8)	46 (19.3)	
25- < 30 years	364 (39.9)	255 (37.8)	109 (45.8)	
≥ 30 years	376 (41.2)	293 (43.4)	83 (34.9)	
**Educational status of mothers** (*n*=905)*				0.339
< high school	362 (40.0)	273 (40.9)	89 (37.4)	
≥ high school	543 (60.0)	394 (59.1)	149 (62.6)	
**Educational status of fathers** (*n*=906)*				0.984
< high school	313 (34.5)	231 (34.5)	82 (34.6)	
≥ high school	593 (65.5)	438 (65.5)	155 (65.4)	
**Occupation of the mothers**				0.401
Employed	474 (51.9)	356 (52.7)	118 (49.6)	
Unemployed	439 (48.1)	319 (47.3)	120 (50.4)	
**Body mass index of enrolled child** (*n*=906)*				0.224
Thinness (< -2 z score)	51 (5.6)	38 (5.7)	13 (5.5)	
Normal (between -2 and 1)	584 (64.5)	423 (63.3)	161 (67.6)	
Overweight (> 1, ≤ 2 z score)	189 (20.9)	150 (22.5)	39 (16.4)	
Obese (> 2 score)	82 (9.0)	57 (8.5)	25 (10.5)	
**Mothers’ perception for enrolled child’s weight** (*n*=911)*				** < 0.001**
Underweight	146 (16.0)	**91 (13.5)**^**a**^	**55 (23.2)**^**b**^	
Normal	672 (73.8)	**519 (77.0)**^**a**^	**153 (64.6)**^**b**^	
Overweight	93 (10.2)	**64 (9.5)**^**a**^	**29 (12.2)**^**a**^	

^a,b^values having different letters in the same row are found to be significantly different

^c^column percentage

*missing data: 8 data for educational status of mothers, 7 for educational status of fathers, 7 for body mass index of enrolled child; 2 for mothers’ perception for enrolled child’s weight

**Fig. 2:**
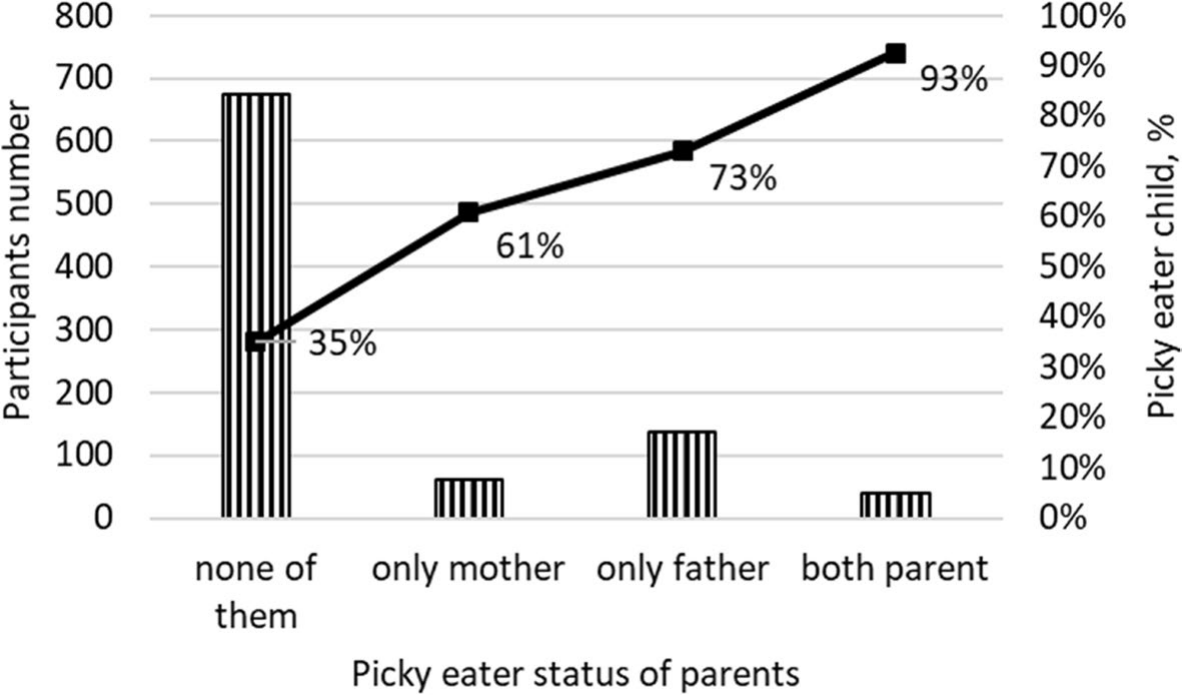
Frequency of picky eating in children according to the parents’ status of picky eating

When the relationship between the parental PE and the child’s PE status was examined; it was found that child's PE increased 3.86 [95% CI: 2.43–6.13] times if the mother was picky eater and 5.77 (95% CI: 3.94–8.47) times increased if the father was picky eater*.* The risk of PE of the child increases 2.85 (95% CI: 1.67–4.88) times when only the mother was picky eater, 5.99 (95% CI: 3.32–7.52) times when only the father was picky eater, and 22.79 (95% CI: 6.95–74.71) times when both of the parents were picky eaters. Figure 2 shows the frequency of PE in children according to the parents’ status of PE.

### Comparison of general characteristics of picky eater children with parental PE status

In the overall group, PE status of the children was compared with the sociodemographic characteristics, and no significant difference was found in terms of province, gender, age, number of children, parental age at birth, paternal educational status. Children of mothers with more than 8 years of education and of working mothers were more picky eaters (*p* = 0.041, *p* = 0.003; respectively). Overweight children were less likely to be picky eaters than thinness, normal weight and obese children (*p* = 0.026). It was observed that the children whose mother perceived as underweight were more picky eaters (*p* = 0.001). It was also found that the children whose parents were picky eaters were more likely to be picky eaters (*p* < 0.001). Table 2 shows the frequencies of children with picky eating according to baseline characteristics in overall group and parents with and without picky eating.

**Table 2:** Frequencies of children with picky eating according to baseline characteristics in overall group and parents with and without picky eating

	**Frequencies of children with picky eating in groups**					
	**Overall group**		**“No parent is picky eater” group**		**“At least one parent is picky eater” group**	
	n(%)^c^	p	n(%)^c^	p	n(%)^c^	p
**Picky eater children**	411 (45.0)		237 (35.1)		174 (73.1)	
**Gender**		0.465		0.994		**0.033**
Female	216 (43.9)		126 (35.1)		**90 (67.7)**	
Male	195 (46.3)		111 (35.1)		**84 (80.0)**	
**Age group**		0.970		0.613		0.947
6–9 years	214 (45.0)		118 (34.2)		96 (73.3)	
10–13 years	197 (45.1)		119 (36.1)		78 (72.9)	
**Number of children in the family**		0.053		0.070		0.662
1	50 (55.6)		29 (48.3)		21 (70.0)	
2–3	316 (44.7)		179 (34.2)		137 874.5)	
≥ 4	45 (38.8)		29 (31.5)		16 (66.7)	
**Age of mother at birth of enrolled child**		0.288		0.040		0.599
< 25 years	124 (41.5)		64 (29.9)^a^		60 (70.6)	
25- < 30 years	171 (46.0)		93 (34.4)^ab^		78 (76.5)	
≥ 30 years	116 (47.9)		80 (41.9)^b^		36 (70.6)	
**Age of father at birth of enrolled child**		0.977		0.942		0.556
< 25 years	79 (45.7)		44 (34.6)		35 (76.1)	
25- < 30 years	164 (45.1)		88 (34.5)		76 (69.7)	
≥ 30 years	168 (44.7)		105 (35.8)		63 (75.9)	
**Educational status of mothers**		0.041		0.211		0.126
< high school	149 (41.2)		89 (32.6)		60 (67.4)	
≥ high school	261 (48.1)		147 (37.3)		114 (76.5)	
**Educational status of fathers**		0.056		0.095		0.236
< high school	127 (40.6)		71 (30.7)		56 (68.3)	
≥ high school	280 (47.2)		163 (37.2)		117 (75.5)	
**Occupation of the mothers**		**0.003**		**0.001**		0.167
Employed	**236 (49.8)**		**145 (40.7)**		91 (77.1)	
Unemployed	**175 (39.9)**		**92 (28.8)**		83 (69.2)	
**Body mass index of enrolled child**		**0.026**		**0.024**		0.755
Thinness (< -2 z score)	**23 (45.1)**^**a**^		**15 (39.5)**^**a**^		8 (61.5)	
Normal (between -2 and 1)	**279 (47.8)**^**a**^		**161 (38.1)**^**a**^		118 (73.3)	
Overweight (> 1, ≤ 2 z score)	**67 (35.4)**^**b**^		**37 (24.7)**^**b**^		30 (76.9)	
Obese (> 2 score)	**40 (48.8)**^**a**^		**22 (38.6)**^**a**^		18 (72.0)	
**Mothers’ perception of child’s weight**		**0.001**		**0.039**		0.402
Underweight	**86 (58.9)**^**a**^		**42 (46.2)**^**a**^		44 (80.0)	
Normal	**285 (42.4)**^**b**^		**177 (34.1)**^**b**^		108 (70.6)	
Overweight	**39 (41.9)**^**b**^		**18 (28.1)**^**b**^		21 (72.4)	
**Picky eating status of the mother**		** < 0.001**				0.962
Yes	**74 (73.3)**				74 (73.3)	
No	**337 (41.5)**				100 (73.0)	
**Picky eating status of the father**		** < 0.001**				**0.011**
Yes	**137 (77.4)**				**137 (77.4)**	
No	**274 (37.2)**				**37 (60.7)**	

^a,b^values having different letters in the same column are found to be significantly different

^c^row percentage

Multivariable logistic regression analysis showed that if mother is employed the child is 1.36 (95% CI: 1.01–1.84) times more likely to be picky eater and if the mother perceives her child underweight, the child is 2.23 (95% CI: 1.30–3.81) times more likely to be picky eater in overall group. In the subgroup in which both parents are not picky eater; it was observed that children were less picky eaters in families with 2–3 or ≥ 4 children than families with a single child [AOR: 0.55 (95% CI 0.32–0.97), AOR:0.48 (95% CI 0.23–0.99); respectively] and it was also found to be that children were more picky eaters in families where the mothers > 30 years old [AOR:1.56 (95% CI 1.00–2.42)]. In the subgroup in which at least one parent was picky eater, it was determined that male children were more picky eaters than their peers [AOR: 2.03 (1.09–3.78)] (Table 3).

**Table 3:** Associations between baseline characteristics and being picky eater children in no parent picky eater group, both picky eater group and overall group^a^

	**Overall group**			**“No parent is picky eater” group**			**“At least one parent is picky eater” group**		
	**AOR**	**95% CI**	**p**	**AOR**	**95% CI**	**p**	**AOR**	**95% CI**	**p**
**Gender, Male vs. female**	1.08	0.83–1.42	0.557	0.98	0.70–1.35	0.878	**2.03**	**1.09–3.78**	**0.025**
**Child’s age, 10–13 vs. 6–9 years**	1.10	0.83–1.44	0.508	1.20	0.87–1.69	0.264	1.03	0.55–1.93	0.940
**Number of children**			0.121			0.091			0.669
**2–3 vs. 1**	0.66	0.42–1.04	0.077	**0.55**	**0.32–0.97**	**0.038**	1.33	0.53–3.33	0.540
**4 vs 1**	**0.55**	**0.30–0.99**	**0.047**	**0.48**	**0.23–0.99**	**0.049**	0.93	0.26–3.31	0.907
**Maternal age, years**			0.611			0.108			0.692
**25–30 vs. < 25**	1.06	0.77–1.46	0.733	1.10	0.74–1.65	0.636	1.23	0.62–2.45	0.559
**> 30 vs. < 25**	1.20	0.83–1.73	0.330	**1.56**	**1.00–2.42**	**0.049**	0.87	0.38–1.99	0.748
**Maternal education duration, ≥ high school vs. < high school**	1.15	0.84–1.56	0.384	0.98	0.67–1.42	0.910	1.42	0.73–2.80	0.305
**Maternal occupation, Employed vs. unemployed**	**1.36**	**1.01–1.84**	**0.042**	**1.52**	**1.06–2.19**	**0.024**	1.54	0.78–3.08	0.217
**Maternal perception for child’s weight**			< 0.001			0.026			0.251
**Normal vs. obese**	1.06	0.68–1.65	0.802	1.33	0.74–2.39	0.334	1.08	0.42–2.78	0.866
**Underweight vs. obese**	**2.23**	**1.30–3.81**	**0.004**	**2.33**	**1.16–4.67**	**0.017**	2.08	0.67–6.48	0.208
**Constant**	0.69		0.296	0.42		0.051	0.82		0.788

*AOR* Adjusted Odds Ratio, *CI* Confidence Interval

^a^multivariable logistic regression

### Comparison of physical activity, screen, sleep and eating habits of all participant children with parental PE status

When the physical activity, screening, sleeping and eating habits of the children in the general group and subgroups were compared, it was found that the frequency of daily consumption of sweet snacks, salty snacks and carbonated beverages was lower in the group in which parents are not picky eater (*p* = 0.005, *p* = 0.011, *p* = 0.005; respectively) **(**Table 4).

**Table 4:** Comparison of physical activity, screen, sleep and eating habits of all participant children with parental picky eating status

	**Overall (*n* = 913)**	**No parent is picky eater (*n* = 675)**	**At least one parent is picky eater (*n* = 238)**	
	n(%)*	n(%)*	n(%)*	p
**Physical activity time of children**				0.182
< 1 h/day	369 (41.0)	264 (39.7)	105 (44.7)	
≥ 1 h/day	531 (59.0)	401 (60.3)	130 (55.3)	
**Screen time of children**				0.421
< 2 h/day	447 (49.2)	326 (48.4)	121 (51.5)	
≥ 2 h/day	461 (50.8)	347 (51.6)	114 (48.5)	
**Sleep time of children**				0.447
< 9 h/day	401 (44.3)	292 (43.5)	109 (46.4)	
≥ 9 h/day	505 (55.7)	379 (56.5)	126 (53.6)	
**Bedtime of children**				0.596
≤ 10:00 pm	572 (64.1)	428 (64.6)	144 (62.6)	
> 10:00 pm	321 (35.9)	235 (35.4)	86 (37.4)	
**Mealtime of children**				0.182
< 20 min	231 (25.3)	175 (25.9)	56 (23.5)	
20–30 min	594 (65.1)	442 (65.5)	152 (63.9)	
> 30 min	88 (9.6)	58 (8.6)	30 (12.6)	
**Having breakfast**				0.478
< 3 day/week	137 (15.0)	98 (14.5)	39 (16.5)	
≥ 3 day/week	774 (85.0)	576 (85.5)	198 (83.5)	
**Skipping meals**				0.433
< 3 day/week	801 (87.9)	596 (88.4)	205 (86.5)	
≥ 3 day/week	110 (12.1)	78 (11.6)	32 (13.5)	
**Mealing at table**				0.431
< 3 day/week	109 (12.0)	84 (12.5)	25 (10.5)	
≥ 3 day/week	801 (88.0)	589 (87.5)	212 (89.5)	
**Mealing in front of screen**				0.054
< 3 day/week	719 (79.4)	542 (80.9)	177 (75.0)	
≥ 3 day/week	187 (20.6)	128 (19.1)	59 (25.0)	
**Snacking at night**				0.400
< 3 day/week	883 (97.4)	656 (97.6)	227 (96.6)	
≥ 3 day/week	24 (2.6)	16 (2.4)	8 (3.4)	
**Having sugary snacks**				**0.005**
< 3 day/week	550 (60.6)	**425 (63.2)**	**125 (53.0)**	
≥ 3 day/week	358 (39.4)	**247 (36.8)**	**111 (47.0)**	
**Having salty snacks**				**0.011**
< 3 day/week	749 (82.2)	**567 (84.1)**	**182 (76.8)**	
≥ 3 day/week	162 (17.8)	**107 (15.9)**	**55 (23.2)**	
**Drinking sugary-carbonated beverage**				**0.005**
< 3 day/week	770 (84.5)	**583 (86.5)**	**187 (78.9)**	
≥ 3 day/week	141 (15.5)	**91 (13.5)**	**50 (21.1)**	
**Drinking ayran**				0.701
< 3 day/week	325 (35.8)	238 (35.5)	87 (36.9)	
≥ 3 day/week	582 (64.2)	433 (64.5)	149 (63.1)	

^*^column percentage

Children who are skipping meals, mealing in front of screen, having sugary snacks, having salty snacks, and drinking carbonated beverage at least 3 day a week were found to be more picky eaters (*p* < 0.001, *p* < 0.001, *p* < 0.001, *p* < 0.001, *p* = 0.003; respectively) in the overall group. In the subgroup in which at least one parent was picky eater, there was also a significant relations of physical activity duration of < 1 h/day, screen time of ≥ 2 h/day and having salty snacks ≥ 3 day/week with being a picky eater (*p* = 0.005, *p* = 0.039, *p* = 0.008; respectively) (Table 5).

**Table 5:** Being a picky eater children according to their physical activity, screen, sleep and eating habits in overall group and groups of no parent and at least one parent picky eating

	**Frequencies of children with picky eating in groups**					
	**Overall group**		**“No parent is picky eater” group**		**“At least one parent is picky eater” group**	
	n(%)^a^	p	n(%)^a^	p	n(%)^a^	p
**Total**	411 (45.0)		237 (35.1)		174 (73.1)	
**Physical activity time of children**		0.082		0.986		**0.005**
< 1 h/day	178 (48.2)		92 (34.8)		**86 (81.9)**	
≥ 1 h/day	225 (42.4)		140 (34.9)		**85 (65.4)**	
**Screen time of children**		0.129		0.307		**0.039**
< 2 h/day	189 (42.3)		108 (33.1)		**81 (66.9)**	
≥ 2 h/day	218 (47.3)		128 (36.9)		**90 (78.9)**	
**Sleep time of children**		0.657		0.486		0.095
< 9 h/day	183 (45.6)		98 (33.6)		85 (78.0)	
≥ 9 h/day	223 (44.2)		137 (36.1)		86 (68.3)	
**Bedtime of children**		0.059		0.152		0.240
≤ 10:00 pm	244 (42.7)		142 (33.2)		102 (70.8)	
> 10:00 pm	158 (49.2)		91 (38.7)		67 (77.9)	
**Mealtime of children**		0.256		0.361		0.093
< 20 min	114 (49.4)		68 (38.9)		46 (82.1)	
20–30 min	256 (43.1)		152 (34.4)		104 (68.4)	
> 30 min	41 (46.6)		17 (29.3)		24 (80.0)	
**Having breakfast**		0.120		0.193		0.588
< 3 day/week	70 (51.1)		40 (40.8)		30 (76.9)	
≥ 3 day/week	340 (43.9)		196 (34.0)		144 (72.7)	
**Skipping meals**		** < 0.001**		**0.001**		0.131
< 3 day/week	**343 (42.8)**		**196 (32.9)**		147 (71.7)	
≥ 3 day/week	**67 (60.9)**		**40 (51.3)**		27 (84.4)	
**Mealing at table**		0.982		0.894		0.757
< 3 day/week	49 (45.0)		30 (35.7)		19 (76.0)	
≥ 3 day/week	361 (45.1)		206 (35.0)		155 (73.1)	
**Mealing in front of screen**		** < 0.001**		**0.008**		0.106
< 3 day/week	**303 (42.1)**		**178 (32.8)**		125 (70.6)	
≥ 3 day/week	**106 (56.7)**		**58 (45.3)**		48 (81.4)	
**Snacking at night**		0.359		0.464		0.906
< 3 day/week	395 (44.7)		229 (34.9)		166 (73.1)	
≥ 3 day/week	13 (54.2)		7 (43.8)		6 (75.0)	
**Having sugary snacks**		** < 0.001**		** < 0.001**		0.438
< 3 day/week	**212 (38.5)**		**123 (28.9)**		89 (71.2)	
≥ 3 day/week	**197 (55.0)**		**113 (45.7)**		84 (75.7)	
**Having salty snacks**		** < 0.001**		**0.006**		**0.008**
< 3 day/week	**313 (41.8)**		**187 (33.0)**		**126 (69.2)**	
≥ 3 day/week	**98 (60.5)**		**50 (46.7)**		**48 (87.3)**	
**Drinking sugary-carbonated beverage**		**0.003**		**0.034**		0.409
< 3 day/week	**331 (43.0)**		**196 (33.6)**		135 (72.2)	
≥ 3 day/week	**80 (56.7)**		**41 (45.1)**		39 (78.0)	
**Drinking ayran**		0.734		0.531		0.111
< 3 day/week	149 (45.8)		80 (33.6)		69 (79.3)	
≥ 3 day/week	260 (44.7)		156 (36.0)		104 (69.8)	

^a^row percentage

Multivariable logistic regression analysis showed that children with skipping meals of ≥ 3 h/day and mealing in front of screen of ≥ 3 h/day were more likely to be picky eaters [AOR:1.99 (95% CI 1.31–3.03), AOR:1.84 (95% CI 1.32–2.57); respectively]. In the subgroup in which at least one parent was picky eater, It was determined that children with physical activity duration of ≥ 1 h/day and sleep time of ≥ 9 h /day were less likely to be picky eater [AOR: 0.42 (95% CI 0.22–0.79), AOR: 0.50 (95% CI 0.26–0.97); respectively]. It was also shown that children with screen time of ≥ 2 h/day were more likely to be picky eaters [AOR: 2.06 (95% CI 1.09–3.90)] (Table 6).

**Table 6:** Risk of being a picky eater children according to their physical activity, screen, sleep and eating habits after controling confounding factors in overall group and groups of no parent and at least one parent picky eating, multivariable logistic regression^a^

	**Risk of being a picky eater children**								
	**Overall**			**No parent is picky eater (*n* = 237)**			**At least one parent is picky eater (*n* = 174)**		
	**AOR**	**95% CI**	**p**	**AOR**	**95% CI**	**p**	**AOR**	**95% CI**	**p**
**Physical activity time of children**									
≥ 1 vs. < 1 h/day	0.81	0.62–1.07	0.135	1.03	0.73–1.44	0.868	**0.42**	**0.22–0.79**	**0.008**
**Screen time of children**									
≥ 2 vs. < 2 h/day	1.24	0.95–1.63	0.119	1.18	0.85–1.65	0.314	**2.06**	**1.09–3.90**	**0.026**
**Sleep time of children**									
≥ 9 vs. < 9 h/day	0.91	0.69–1.21	0.913	1.16	0.82–1.63	0.397	**0.50**	**0.26–0.97**	**0.042**
**Bedtime of children**									
> 10:00 vs. ≤ 10:00 pm	1.26	0.94–1.69	0.118	1.19	0.83–1.69	0.341	1.47	0.75–2.90	0.265
**Mealtime of children**			0.203			0.172			0.111
20–30 vs. < 20 min	0.75	0.55–1.03	0.075	0.79	0.54–1.15	0.215	0.46	0.21–1.01	0.054
> 30 vs. < 20 min	0.79	0.48–1.31	0.363	0.55	0.28–1.06	0.074	0.81	0.25–2.61	0.728
**Having breakfast**									
≥ 3 vs. < 3 day/week	0.70	0.48–1.02	0.064	0.70	0.44–1.10	0.124	0.73	0.31–1.73	0.472
**Skipping meals**									
≥ 3 vs. < 3 day/week	**1.99**	**1.31–3.03**	**0.001**	**2.22**	**1.35–3.64**	**0.002**	2.12	0.74–6.12	0.163
**Mealing at table**									
≥ 3 vs. < 3 day/week	0.92	0.60–1.39	0.684	0.95	0.58–1.56	0.830	0.73	0.27–2.01	0.545
**Mealing in front of screen**									
≥ 3 vs. < 3 day/week	**1.84**	**1.32–2.57**	** < 0.001**	**1.68**	**1.12–2.53**	**0.012**	2.00	0.93–4.30	0.077
**Snacking at night**									
≥ 3 vs. < 3 day/week	1.47	0.64–3.38	0.366	1.52	0.55–4.26	0.421	1.58	0.27–9.21	0.610

*AOR* Adjusted Odds Ratio, *CI* Confidence Interval

^a^Controlled for gender and age of children, number of children, maternal age, education, maternal occupation, maternal perception for child's weight

## Discussion

In this study, the frequency of PE in Turkish children was found to be as high as 45.0%. Although there is no study examining the prevalence of PE in Turkish children in the literature, according to a study conducted for the problematic eating behaviors in children 12–74 months, in Turkey, 39% of mothers stated that their children were picky eater [16]. Due to the differences in the definition of PE and methodological differences of the studies, the frequency and prevalence of PE has been reported to be in a wide range in different studies [25]. In their review, Samuel et al. divided the studies into 3 groups according to the way children questioned and reported the prevalance ranges of PE between (i) 12.3%-49% in 3 studies using 'one closed-ended question’; (ii) 6.6%-59.3% in 14 studies using ‘a single question selected from predetermined possible answers’ (iii) 5.5%-70.1% in 21 studies using ‘several question and answer combinations’ [26].

Previous studies showed that non-responsive parental feeding practices have been associated with greater PE in children [27]. Practices such as pressuring the child to eat healthy foods or using the child's favorite foods as a bribe have been associated with PE in children [28, 29]. Besides nutritional practices of families on children’s eating behaviors, genetic influences were also examined in recent studies. Cooke et al. investigated the relations of neophobia with genetic and environmental influences in 5390 twin children aged 8–11 years and found high familial inheritance. Based on this finding, the researchers suggested that resistance to new foods in children is related not only to parental malpractice but also to genetic inheritance [30]. Furthermore, a new study showed that picky eating behavior was greatly presented in children of lower educated mothers or heavy-smoking fathers [31]. Although some parental characteristics of picky eater children were examined in the literature, there is no study investigating the relations of PE in children with parental PE. We found that the risk of PE in children increases 2.85 times if only the mother was picky eater, 5.99 times if only the father was picky eater, and 22.79 times if both of the parents were picky eaters. There is a need for new studies investigating these relations and enlightening the environmental and genetic aspects of this relevance.

Results of the previous literature on the relationship between PE and sociodemographic characteristics differs. Steinsbekk et al. found no relationship with gender in their cohort study [1], but Cardona Cano et al. found that PE status persisted more in boys [32]. Taylor et al. reported the sociodemographic conditions that increase the risk of PE as being male gender, older maternal age, higher maternal education and lower parity [7]. Sibling presence was also found to be protective [33]. We found that the risk of PE increases in the children of working mothers and older mothers in the group ‘non of the parents are picky eater’, and the risk of PE decreases in children having sibling. We also showed that boys were 2.03 times more picky eater when at least one of the parents were picky eater. This relationship may be due to the fact that boys are more affected by the eating characteristics of their parents. Studies in which only girls were investigated found that mothers' positive eating habits decrease the risk of PE in girls [34]. Parental influence on boys should be investigated with future similar studies.

The relationship between children's growth and PE has been examined in many studies. Some studies found that picky eater children were more likely to be underweight [35–37], some found picky eater children were less likely to be overweight or obese [34, 38], while others found no such relationship [39–41]. The reason for these different results may be the methodological differences of the studies. We found that overweight children were less likely to eat picky than their peers. However, we did not find a similar association for those who were obese. This may be because obese children have an increased tendency to eat high-calorie fast food, which makes them more picky. We also found that the mother’s perception of the child as underweight increases the child’s PE. This relationship may be due to mothers perceive the child as underweight because of the child is a picky eater or the child’s picky due to the forcing of the mothers who perceive their child underweight. In recent studies; it has been reported that parents who think their child is not growing enough are more likely to overreact when their child’s food intake changes [42], and excessive anxiety about the feeding of their child in mothers causes inappropriate and oppressive feeding practices [43, 44].

A limited number of studies investigating the physical activity and screen time habits of children who are picky eaters include only preschool children. In the study by Sandvik et al., parents of picky eaters reported that their children had too much screen time, complained about physical activity, and expressed a negative attitude towards eating [13]. Chao also reported higher levels of insufficient physical activity in preschooler picky eaters [18]. Li et al. found that both picky eaters and children whose daily screen time reached 1 h/day were more likely to consume sugary foods and drink sugar-sweetened beverages [45]. While there are no studies in the literature investigating the relationship between picky eating and screen time in schoolchildren, a recent study of schoolchildren reported that the longer the screen time the increased the odds of unhealthy dietary habits such as skipping breakfast, consuming fast food frequently, and eating sweets frequently [46]. To the best of our knowledge, there is also no study in the literature directly investigating the relationship between sleep habits and PE. However, previous studies have found that adequate sleep is positively associated with a healthy dietary habit among children, adolescents, and adults, and insufficient sleep is inversely associated with health-related behaviors. It was shown that insufficient sleep was associated with negative and binge eating behaviors [14, 47, 48]. Delahunt et al. also observed an inverse associations between sleep duration and ‘Emotional Undereating’ and ‘Food Fussiness’ in 5 year old children [49]. Present study found that PE in children was associated with less daily physical activity and less daily sleep time, and more daily screen time, but this association was only observed when at least one parent was picky eater. The emergence of this relationship, especially when parents are picky eaters, suggests that parental role models are highly determinative in children's lifestyle behaviors.

The strength of this study is that it is the first study to show the relationship between PE in children and parents, and in this context, to reveal the relationship between PE and various demographic factors and health-related behaviors of children. There are also some limitations in this study. The first limitation is that it was conducted in only two provinces. This study showed that the prevalence of PE in the two provinces was similar and also provided information about the associated characteristics. There is a need for new studies with a larger sample, the results of which can be attributed to the whole of Turkey. The second limitation is that the anthropometric measurements of the children were reported by the parents in this study. It has been reported that height and weight reported by the child or parent are generally used in population surveys because they are easy to collect, but these estimated values may be misreported due to systematic differences between sociodemographic characteristics [50].

## Conclusion

This study showed that PE is a common eating problem and it is important to document its relationship with parental characteristics. For the first time, the strong relationship between parental and children’s PE habit has been demonstrated with this study. Whether the origin of this relationship is genetic or environmental is a new research topic for future studies. It was also observed that PE in children was associated with lifestyle habits in the presence of the parental picky eating. Our study revealed that parental influence should be taken into account in attempts to develop healthy eating behaviors and lifestyle habits of children.

## Data Availability

Data can be requested from corresponding author (siyalcin@hacettepe.edu.tr).

## Abbreviations

AOR: Adjusted Odds Ratio
BAZ: Body mass index for age z-scores
BMI: Body mass index
CI: Confidence interval
PE: Picky eating
WHO: World Health Organization
